# An ex vivo gut mucosal explant assay to compare HIV tissue susceptibility shows increased HIV susceptibility with methamphetamine exposure

**DOI:** 10.1186/s12977-026-00684-7

**Published:** 2026-08-18

**Authors:** Julie Elliott, Nicholas E. Webb, Paola Ruiz, Grace D. Cho, Ayub Ali, Peter A. Anton, Otto O. Yang, Jennifer A. Fulcher

**Affiliations:** 1 Division of Infectious Diseases, Department of Medicine, UCLA David Geffen School of Medicine, Los Angeles, CA USA; 2 Ragon Institute of Mass General, MIT, and Harvard, Cambridge, MA USA; 3 Division of Digestive Diseases, Department of Medicine, UCLA David Geffen School of Medicine, Los Angeles, CA USA

**Keywords:** HIV, Mucosal transmission, Methamphetamine, Gut explant, Infection susceptibility

## Abstract

**Background:**

Existing ex vivo mucosal explant models for HIV assess viral replication but do not fully capture the ability of tissue to support productive infection and transfer to target cells, an important indicator of tissue susceptibility. Additional tools to investigate relative mucosal susceptibility to HIV are necessary to better understand risk factors, such as substance use, or to evaluate therapeutics.

**Results:**

We developed an ex vivo gut mucosal explant assay incorporating a CCR5/CXCR4-expressing GFP-indicator cell co-culture to quantify both viral replication and transfer. Susceptibility was quantified using a composite metric based on time to infection endpoint across viral doses. Modulators of susceptibility, including anti-CD3 stimulation, tenofovir, and methamphetamine, were evaluated. The assay reliably detected differences in tissue susceptibility under various experimental conditions. We detected enhanced susceptibility following anti-CD3 stimulation, while tenofovir pretreatment reduced susceptibility in a dose-dependent manner. Methamphetamine exposure resulted in a modest but significant increase in gut tissue susceptibility to HIV.

**Conclusions:**

This novel ex vivo explant susceptibility assay advances HIV mucosal transmission research by measuring both viral production and transfer. It can detect subtle changes in susceptibility, providing a versatile platform for evaluating prevention therapeutics, host and microbial factors, and substance use effects.

**Supplementary Information:**

The online version contains supplementary material available at https://doi.org/10.1186/s12977-026-00684-7.

## Background

Mucosal transmission of HIV is the predominant source of newly acquired infections, and rectal transmission accounts for over 60% of new infections in the United States [1]. While most cases of rectal transmission occur in men who have sex with men (MSM), it is an underappreciated source of transmission in heterosexual women [2], making it a phenomenon with broad public health implications.

Methamphetamine use has been repeatedly associated with increased HIV incidence [3–6]. Both behavioral and biological factors likely mediate the association between methamphetamine exposure and HIV transmission. Prior studies have shown that methamphetamine can increase expression of HIV co-receptors (CCR5 and CXCR4) and enhance viral replication in CD4 + T cells [7] and myeloid cells [8, 9]. At least two clinical studies showed increased inflammatory cytokine expression in the rectal mucosa after methamphetamine use [10, 11]; to date no studies have examined the direct impact of methamphetamine on gut tissue HIV infection.

Ex vivo gut explant infection models use intact human colorectal tissue with the goal of maintaining cellular architecture and immune microenvironments to study early events of mucosal HIV infection. These models have been used extensively in the pre-clinical testing of HIV prevention therapeutics [12]. Prior studies have shown that colorectal explants contain comparable percentages of CD4 + and CD8 + T cells as peripheral blood, though a greater percentage of CD4 + T cells are CCR5 + and CD38 + HLA-DR+ activated phenotype in the gut. Multiple studies have also shown that the relative percentages of CD4 + and CD8 + T cells remain grossly stable over 7–10 days in explant culture [12, 13]. We previously found that absolute CD4 + T cell counts in colorectal explants varied as much as 10-fold across individuals, though the relative proportion of activated CD4 + T cells was comparatively more stable between donors [14]. Colorectal explants represent an important tool for understanding factors which regulate HIV replication and latency [13, 15] or alter susceptibility to infection [16]; however, standardized methods to compare infection susceptibility across tissues are limited.

Current ex vivo explant models typically use cumulative p24 antigen measured by ELISA or HIV gag measured by quantitative real time PCR as infection endpoints [16–18]. These measures quantify viral particle production but do not assess the ability of tissue to produce and transfer productive virus, which is necessary for sustained infection. Further, current models often employ supra-physiologic doses to ensure tissue infection, which limits the ability to compare infectivity across tissues in a physiologic context.

We sought to develop an ex vivo explant assay that can quantify relative HIV susceptibility in gut tissues. We approached this by focusing on the ability of gut tissue to support productive viral replication and transfer to susceptible target cells. Here we describe a co-culture system using a limiting dose titration approach with indicator cells to compare relative tissue susceptibility in colorectal tissue explants, and use this method to examine the effects of methamphetamine exposure of gut tissue HIV susceptibility.

## Methods

### Tissue specimens and explant generation

Intestinal tissues used in this study were obtained deidentified from the Translational Pathology Core Laboratory (TPCL), a research facility in the Department of Pathology at the University of California Los Angeles. Normal appearing remnant tissue specimens procured from the large intestine were released for research purposes following pathologist review. Specimens, varying in size from 3 cm to 10 cm^2^, were collected into 25–30 ml of RPMI1640 and stored at 4 degrees until transport to the laboratory (generally between 1 and 12 h post-surgery). Upon receipt in the laboratory, tissues specimens were washed 3–5 times in sterile PBS and transferred to a petri dish containing explant media (RPMI 1640 supplemented with 10% FBS (Atlanta Biologicals), 1x antibiotic-antimycotic solution (Gibco cat#15240062, Fisher Scientific), and 0.1 mg/ml piperacillin-tazobactam (Pfizer, New York, NY). The mucosa was dissected from the sub-mucosa using sterile scissors and 4 mm explants were generated using disposable dermal biopsy punches (Integra Miltex cat#12-460-409, Fisher Scientific). Explants were cryopreserved and subsequently thawed according to the protocols detailed by Hughes et al. [19]. Prior to use, explants were rapidly thawed and transferred using a sterile pipet to a petri-dish containing 15–20 ml of explant media for equilibration at room temperature for 10–15 min before further use.

### HIV-1 virus expansion

HIV-1_Bal_ was obtained from the NIH AIDS Reagent Program, Division of AIDS, National Institute of Allergy and Infectious Diseases (NIAID). Freshly isolated peripheral mononuclear cells (PBMC) were provided by the UCLA CFAR Centralized Laboratory Support Core then CD4 + T cells were expanded using a CD3-CD8 bispecific antibody (BEI Resources/NIH AIDS Reagent Repository catalog # ARP-12277 contributed by Drs. Johnson Wong and Galit Alter) in the presence of 50U/ml of IL-2. Following 5–6 days of expansion, cultures comprised > 90% CD4 + T cells as verified by flow cytometry. Stocks of HIV-1_BaL_ were generated following incubation for 2–4 h in 1 ml of virus stock and subsequent tissue culture. Virus containing supernatants were harvested between days 6 to 9 and the TCID50 determined by Reed-Muench method using activated PBMC. The same viral stock was used throughout this study.

### Indicator cells

T1-CCR5 cells are an X4-HIV-1 permissive immortalized CD4-expressing lymphocyte cell line [20, 21] that was stably transduced with CCR5 [22]. These cells were then transfected with at Tat- and Rev-dependent eGFP expression gene kindly provided by Dr. Benhur Lee [23]. Limiting dilution cloning was performed to enrich for a pure population with high eGFP expression after HIV-1 infection.

### Explant infection and indicator cell co-culture

Explants were thawed and equilibrated as described above. Triplicate explants were added to wells of a 24-well tissue culture plate containing 1 ml of complete explant media. Agents proposed to either enhance or reduce mucosal HIV susceptibility were added: anti-human CD3 antibody (12F6 clone, generous gift from Dr. Johnson Wong) 200ng/ml with 50U/ml IL-2 (PeproTech, ThermoFisher cat#200-02-1MG), 10 μm–1 μm tenofovir disoproxil fumarate (NIH HIV Reagent Program), 100 μm methamphetamine (Sigma cat#M8750), or control (media alone). Methamphetamine dose was based on prior in vitro studies as well as measured tissue concentrations in animal models [24–26]. Cultures were incubated overnight at 37 °C /5% CO2 in a humidified tissue culture chamber. The following day, freshly thawed HIV_Bal_ was added to the explant cultures at limited dilutions with final TCID50 of 5 × 10^4^, 5 × 10^3^, or 5 × 10^2^ then incubated at 37 °C for 2 hours. Pilot experiments included an additional negative control condition with TCID50 5 × 10^4^ heat-inactivated HIV_Bal_. Explants were then transferred to fresh wells containing 1-2 ml of explant media and washed thoroughly 5 times by complete media exchange. Washed explants were then individually added to wells of a 24-well plate containing 1 × 10^5^ T1-CCR5-GFP cells in 1 ml explant media. Co-culture wells were sampled at daily intervals by the removal of 100ul of media, after ensuring that cells were well resuspended. Cells were transferred to a round bottom tissue culture plate and centrifuged at 400 g for 5 minutes in sealed buckets and then fixed by resuspension in 2% paraformaldehyde. Amount of infection (%GFP) was analyzed using a FACSymphony A1 cytometer (Becton Dickenson, San Jose, CA) equipped with a high throughput adaptor. Cells were gated using forward and side scatter and the percentage of GFP expression was quantified as compared to an uninfected T1-CCR5-GFP cell control. A minimum of 2000 events were analyzed.

### Explant mucosal cell isolation and flow cytometry

Mucosal mononuclear cells were isolated from colorectal explants using collagenase digestion as previously described [27]. After washing cells were counted and viability assessed using trypan blue dye exclusion. Cells were then stained for flow cytometry with the following antibodies (BioLegend, San Diego, CA): CD45-PerCP-Cy5.5 (clone 2D1), CD3-Pacific Blue (clone UCHT1), CD4-APC (clone SK3), CD8-APC-Fire750 (clone SK1). After cell surface staining cells were fixed and permeabilized using Cytofix/Cytoperm (Becton Dickenson, San Jose, CA) then stained for intracellular p24 antigen with KC57-PE antibody (Beckman Coulter, Brea, CA). Cells were analyzed using a FACSymphony A1 cytometer (Becton Dickenson, San Jose, CA) with a minimum 10,000 events acquired. Analysis was performed with FlowJo v10.10.1 (Becton Dickenson, San Jose, CA). Gating is described in Supplementary Fig. S1.

### Statistical analyses

Time to infection endpoint was interpolated from T1-CCR5-GFP time course data using Python 3.6 (scipy.interpolate). The area under the curve (AUC) from the interpolated time to infection vs. TCID50 dose graph was calculated using Python 3.6 (numpy.trapz). Comparisons between treatment conditions and/or groups were analyzed using paired or one-sample t-tests or Wilcoxon signed rank tests as appropriate. Data visualization and analyses were performed using GraphPad Prism v10.6.1.

## Results

### Development of tissue susceptibility protocol

**Fig. 1:**
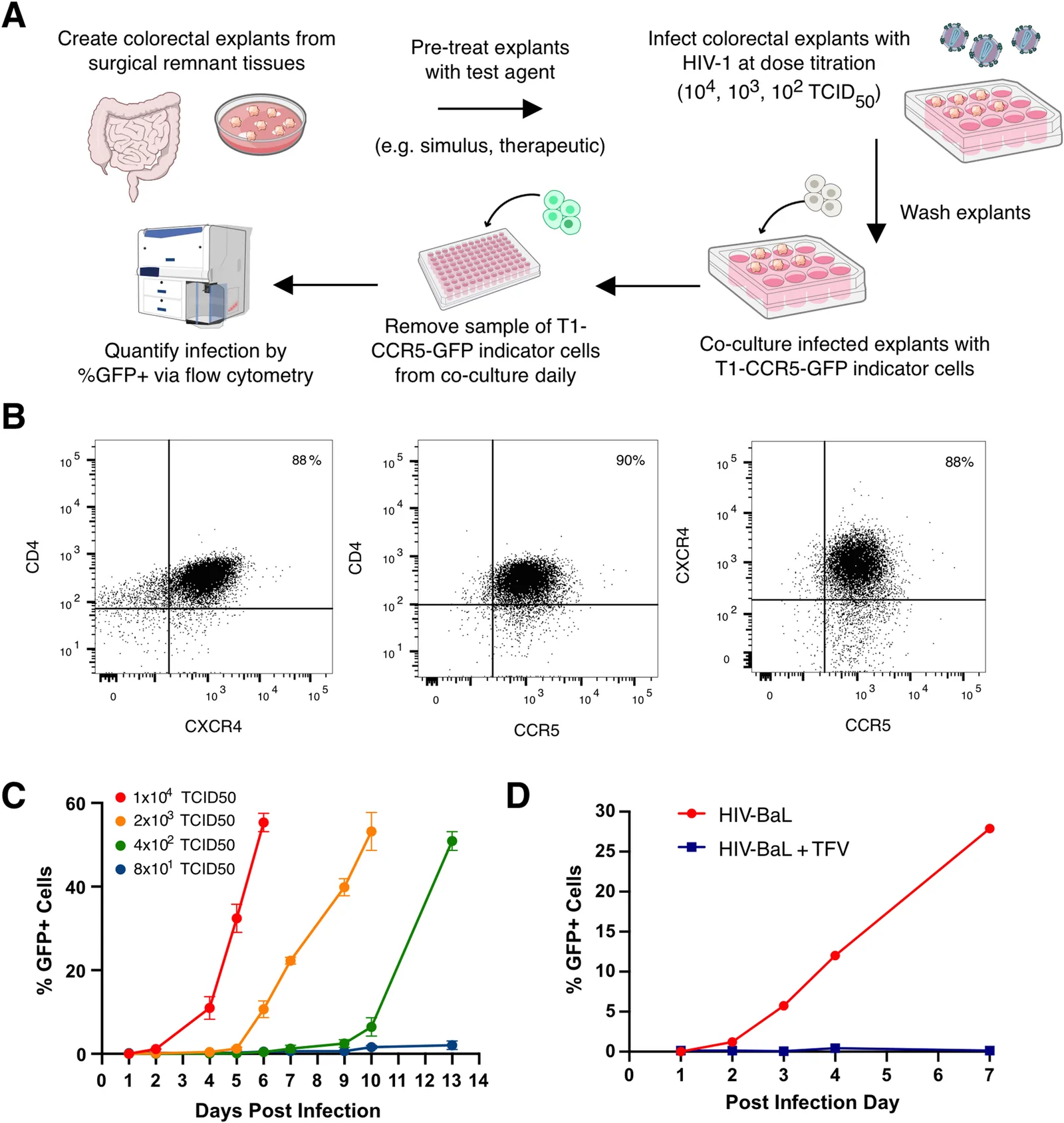
Ex vivo gut tissue HIV susceptibility assay overview. **A** Assay schematic using gut explants and T1-CCR5-GFP indicator cells to measure productive infection. Illustrations from NIAID NIH BioArt Source. **B** Flow cytometry showing CD4 plus CCR5 and CXCR4 co-receptor expression on generated T1-CCR5-GFP indicator cells. These cells have endogenous CXCR4 expression and were stably transduced to have CCR5 expression. Right panel shows dual co-receptor expression on all cells. **C** Infection kinetics of T1-CCR5-GFP indicator cells at varying TCID50 doses. Infection measured by %GFP positive with point indicating mean value of duplicate cultures and error bars indicating standard deviation. Infection was detectable as early as two days post-infection and, as expected, lower doses were not detected until later days. **D** Infection kinetics showing no detectable infection in the presence of 10 μm tenofovir (blue) compared to 10^4^ TCID50 HIV-BaL (red)

To quantify relative tissue susceptibility, we created a measure that would include the ability of infected tissue to produce and transfer virus to adjacent cells rather than simply quantify viral nucleic acids or proteins. In addition, we wanted to include limiting dilutions of virus to better mimic physiologic conditions where differences in tissue infection may be discernable. To do this, we developed a protocol where explants were infected with three TCID50 doses and infection was monitored by a highly susceptible GFP-indicator cell line that would amplify early virus, thereby making early stages of explant infection easier to detect (Fig. 1A). The indicator cell line was T1-CCR5-GFP cells, an immortalized CD4 + lymphoblastoid cell line containing a Tat/Rev-dependent eGFP indicator gene for HIV infection. In addition to the GFP reporter, these cells had endogenous CXCR4 expression and stably transduced CCR5 expression, allowing for use with both X4 and R5 viruses (Fig. 1B). T1-CCR5-GFP cells were readily infected with HIV at multiple TCID50 doses with infection detectable as early as two days post-infection (Fig. 1C). The limit of detection for the T1-CCR5-GFP indicator cells was at least as low as 4 × 10^2^ TCID50 dose, with no detectable infection at the 8 × 10^1^ TCID50 dose. Infection was inhibited in the presence of tenofovir (Fig. 1D), even at highest infectious dose (1 × 10^4^ TCID50).

### Infection kinetics differs by input dose and tissue stimulation

**Fig. 2:**
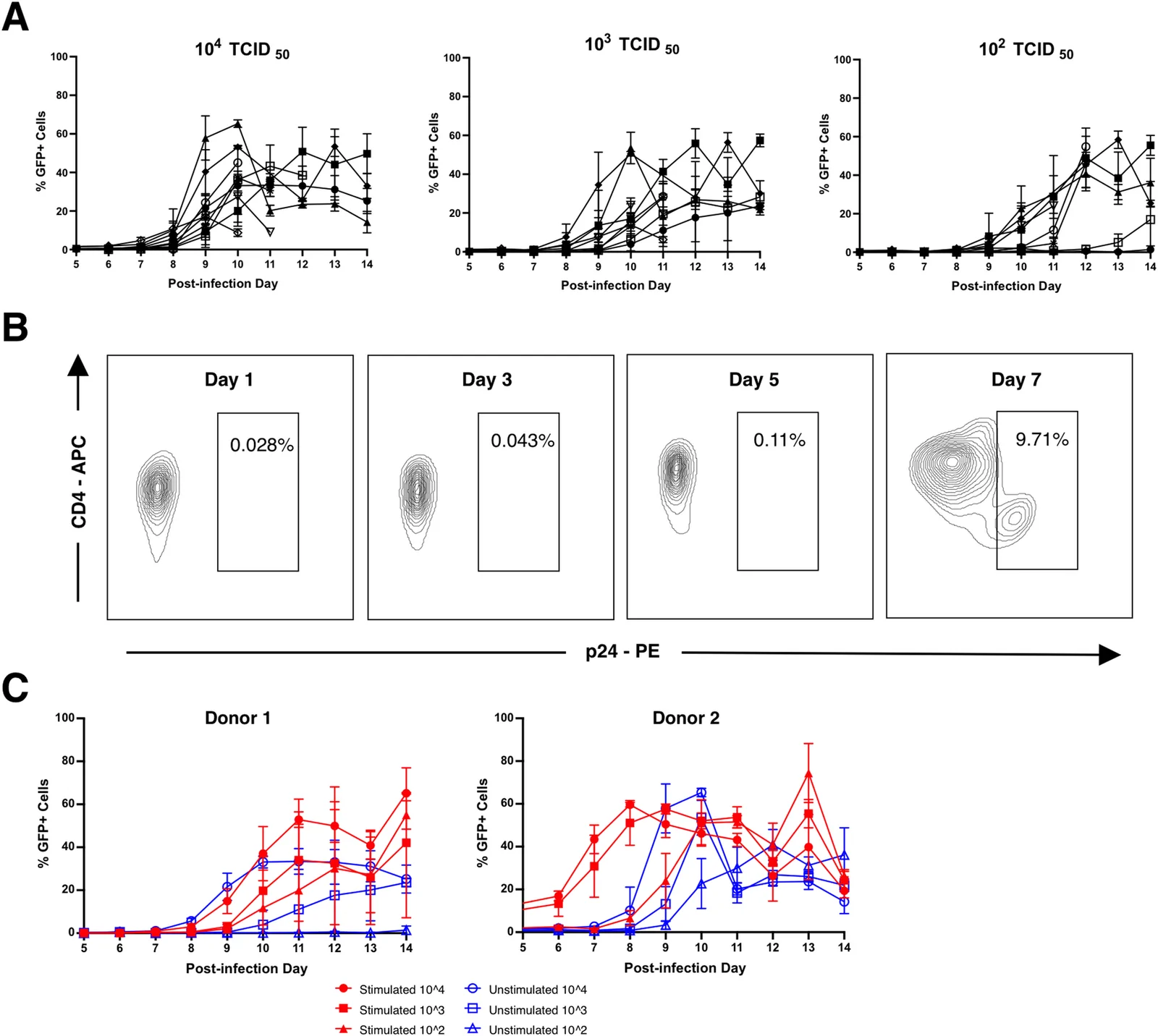
Variation in HIV infectability by virus dose and stimulation. **A** Infection kinetics in explant co-culture system at different TCID50 doses. Infection measured daily and indicated by %GFP positive in T1-CCR5-GFP indicator cells. Each symbol represents an individual donor; the same 9 donors are included in all three TCID50 doses. Symbols represent the mean % GFP-positive cells from triplicate explant infections with error bars representing standard deviation. Plots show that at higher doses infection is detectable as early as six days post-infection with lower doses not detectable until later days. **B** Flow cytometry showing intracellular p24 staining in explant-derived CD4 + T cells at indicated days post-infection. Infection is detectable by day 7 in explant cells, which correlates with time course of indicator cells in our co-culture system. **C** Infection kinetics in explant co-culture system with (red) anti-CD3 pre-stimulation or without (blue) stimulation. Symbols represent the mean % GFP-positive cells from triplicate explant infections with error bars representing standard deviation. Plots show earlier detectable infection in stimulated explants

The infection kinetics in our co-culture system across doses is shown in Fig. 2A. As expected, higher TCID50 resulted in early detectable infection with lower TCID50 resulting in later detectable infection. Infectability differed by dose so using dose titration composite may give a better assessment of tissue susceptibility. In addition, there was great variability over the course of infection and between individuals. To confirm we were detecting true infection of the explant tissue, we also performed intracellular p24 staining on mucosal mononuclear cells isolated from the explants. As shown in Fig. 2B, we observed infection in explant-derived mucosal CD4 + T cells at time points that correlated with our co-culture results, suggesting that our co-culture system accurately represented infection derived from the explant. In addition, the time course of direct infection of the T1-CCR5-GFP cells (Fig. 1C) was much earlier than detectable explant co-culture infection, indirectly validating that our co-culture system detects infection derived from the explant. Notably, we could only reliably observe mucosal CD4 + T cell infection by flow cytometry at higher (1 × 10^4^ TCID50) doses, suggesting that our GFP-indicator cell readout is a more sensitive measure than intracellular p24 flow cytometry. As expected, there was a decrease in cell viability over time in explant culture, but relative percentages of CD4 + T cells were stable which is consistent with prior studies [12, 13] (Supplementary Fig. 1).

We next increased tissue susceptibility by pre-treating explants with anti-CD3 stimulation with IL-2, then assessed our ability to distinguish differences. As seen in Fig. 2C, stimulated explants showed an earlier and more rapid increase in measurable T1-CCR5-GFP cell infection. Therefore, our assay can detect differences in infection with or without stimulation, and the greatest differences appear early in the infection.

**Fig. 3:**
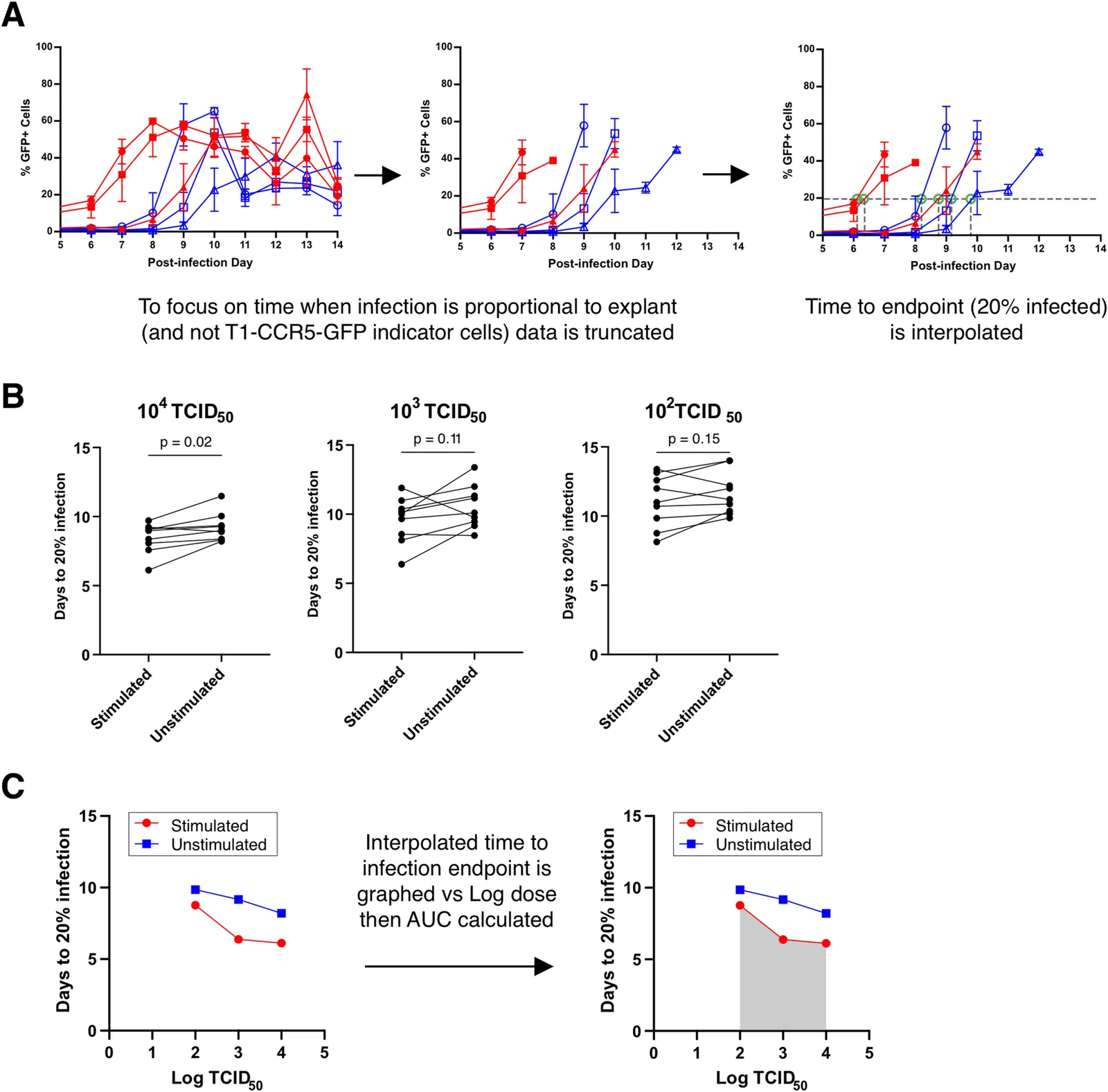
Analytic strategy and composite metric for comparative susceptibility. **A** Schematic depiction of infection endpoint interpolation. Endpoint was chosen at 20% GFP + as this was the point when most infections entered the early burst phase of growth. **B** Comparison of interpolated time to infection by TCID50 dose. Each pair of points represents an individual donor. *P*-values calculated using paired *t*-tests. **C** Schematic depiction of composite area under the curve susceptibility metric

### Relative susceptibility metric can reliably distinguish changes in HIV susceptibility

**Fig. 4:**
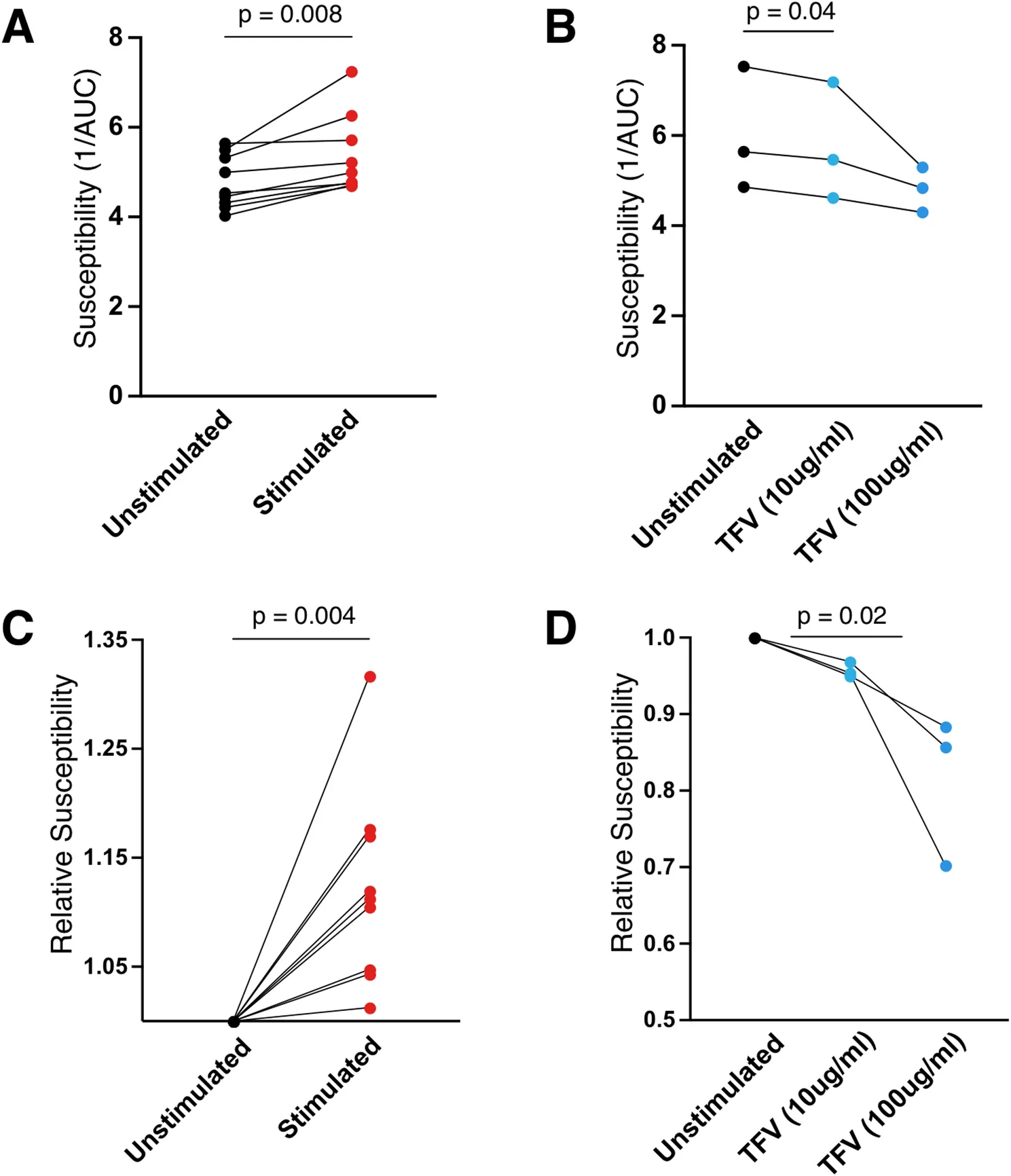
Ex vivo explant susceptibility assay can detect increases and decreases in gut tissue HIV susceptibility. **A** Increased susceptibility following anti-CD3 stimulation or (**B**) decreased susceptibility following tenofovir (TFV) treatment detected by explant susceptibility assay using composite AUC metric. Each paired point represents an individual donor. *P*-values determined using paired *t*-tests. The same data are also represented as relative susceptibility compared to untreated condition for (**C**) anti-CD3 stimulation and (**D**) tenofovir treatment. Each paired point represents an individual donor. *P*-values determined using Wilcoxon signed rank test for C or one-sample *t*-test for D.

In order to compare susceptibility between individuals and/or treatment conditions, we created a metric that incorporated data from all dose titrations. To ensure analysis focused on virus transferred from the explant and not ongoing viral replication in the T1-CCR5-GFP cells, we focused only on the initial phase of infection based on our data (Fig. 2). We reasoned that tissues that reached this end point more quickly were more susceptible to infection, and that end point analysis would more reliably reflect the efficiency of the iterative process of infection compared to final amount. Thus, we interpolated the time to infection endpoint defined as 20% GFP-indicator cell infection (Fig. 3A). This endpoint was chosen as this was the point when most infections had entered the early burst phase of growth. When comparing the time to infection endpoint between stimulated and unstimulated tissues, we found inconsistent differences by infection dose (Fig. 3B). This underscores the importance of using a titration approach to fully assess the tissue susceptibility. In order to incorporate data from all TCID50 dose titrations, we used the area under the curve (AUC) for comparisons (Fig. 3C). As shown in Fig. 4A, we could reliably detect differences in tissue susceptibility between stimulated and unstimulated conditions in a variety of individuals. We also assessed our ability to detect decreases in susceptibility by pre-treating explants with different doses of the antiretroviral drug tenofovir. As shown in Fig. 4B we could reliably detect a dose-response decrease in tissue susceptibility with tenofovir using our assay. To make the susceptibility measure more interpretable as a comparative metric, we normalized to the unstimulated condition showing the ability to detect up to 30% differences in susceptibility (Fig. 4C, D). We also compared susceptibility within our tissue donor pool by age and by sex but found no significant differences (Supplemental Fig. S1) in this small sample group.

**Fig. 5:**
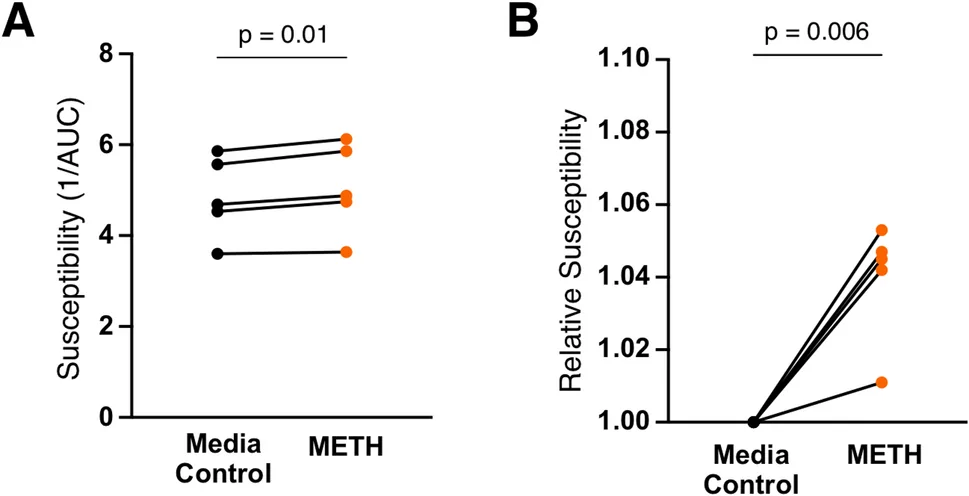
Methamphetamine exposure increases gut tissue HIV susceptibility. **A** Increased susceptibility following methamphetamine (METH) exposure. Each paired point represents an individual donor. *P*-value determined using paired *t*-tests. **B** The same data is also represented as relative susceptibility compared to untreated condition following methamphetamine exposure. Each paired point represents an individual donor. *P*-value determined using one-sample *t*-test

### Methamphetamine increases HIV susceptibility in gut tissue

To begin to address a long-standing question regarding the biological effects of methamphetamine on HIV transmission, we examined the direct effects of methamphetamine exposure on HIV susceptibility using our assay. We found that methamphetamine exposure resulted in a small, but significant, increase in HIV susceptibility (Fig. 5). Coupled with behavioral factors, this measurable change in tissue susceptibility could help explain the increased risk of HIV mucosal transmission in people who use methamphetamine.

## Discussion

Here we describe a novel ex vivo gut mucosal explant assay to quantify relative tissue susceptibility to HIV infection. This assay goes beyond standard colorectal explant models in that it assesses both virus production and subsequent transfer using a GFP-indicator cell readout. This readout provides a more nuanced assessment of mucosal susceptibility, focusing not only the capacity of tissue to support viral replication but also the efficiency of transmission to target cells. We showed the ability to detect both increases and decreases in relative susceptibility, and used this method to show that methamphetamine can directly increase gut tissue susceptibility to HIV infection.

These findings align with epidemiological data linking methamphetamine use to increased HIV acquisition risk [28, 29], and provides evidence for a biologic mechanism linking substance use and mucosal HIV susceptibility. Previous studies have implicated immune activation as a potential mediators of methamphetamine-associated HIV risk [8, 9]. Our study is the first to provide direct ex vivo evidence that methamphetamine can act directly on gut mucosal tissue to enhance susceptibility. While these experiments showed potential direct effects of methamphetamine, additional studies are needed to validate these findings and further investigate factors such as duration of the effect, dose-response relationship, and the underlying mechanisms.

A strength of this assay is the ability to detect both subtle and larger increases and decreases in susceptibility. This method could have broad utility investigating host, microbial, and environmental factors that shape mucosal HIV transmission dynamics. The intestinal microbiome is increasingly recognized as a key determinant of mucosal function and inflammation in HIV [30, 31], and more recently has been implicated in HIV susceptibility risk [32, 33]. Our assay could be used to evaluate the role of microbiome-derived metabolites or microbial communities in human tissue permissiveness to HIV. In addition, this assay can easily be applied to pre-clinical testing for prevention therapeutics including microbicides or small molecule inhibitors, where quantitative comparisons of relative susceptibility across condition are important.

This method also could be used to examine population-level differences in susceptibility, though the degree of donor variability inherent in human mucosal explants would necessitate a large sample size to detect significant differences. For this study, since all comparisons were relative, any individual differences in target cell numbers, host factors, or processing variations were likely minimized. However, these are important variable to consider for population-level studies. Nonetheless, the reproducibility of the assay and the ability to detect directional changes make it a potentially valuable comparative tool. Future adaptation for higher-throughput screening using plate-based flow cytometry would increase scalability and enable larger studies of determinants of HIV susceptibility.

This study has several limitations. First, ex vivo explant models, while more physiologically relevant than cell line systems, cannot fully recapitulate the dynamic environment of the human gut, including factors such as luminal microbiota, vascular flow, and immune cell trafficking. In addition, there is known deterioration in tissue architecture and decreased viability over time in culture despite optimal culture conditions [12, 13], and this could contribute to variability in infection. Effects from variations in processing timing and/or cryopreservation is also a potential consideration [19]. Second, our assay focuses primarily on productive infection and transmission within a controlled ex vivo context, and therefore may not capture the full spectrum of host-pathogen interactions that occur in vivo. Finally, while the assay provides relative susceptibility readouts, it does not directly quantify absolute infection risk.

## Conclusions

Our ex vivo gut mucosal explant susceptibility assay enables simultaneous assessment of virus production and transfer, offering a powerful platform to investigate how drugs, microbes, and host factors alter tissue susceptibility. Using this assay we showed that methamphetamine exposure induces a measurable increase in gut mucosal tissue infection; an important finding that helps explain the clinical association between methamphetamine use and increased HIV acquisition. This assay system could be adapted for HIV prevention research, therapeutic development, and to enhance our understanding of the mucosal determinants of HIV acquisition.

## Supplementary Information

Below is the link to the electronic supplementary material.

Supplementary Material 1.

## Data Availability

The datasets used and/or analyzed during the current study are available from the corresponding author on reasonable request.
