# Supplementary material for: An ex vivo gut mucosal explant assay to compare HIV tissue susceptibility shows increased HIV susceptibility with methamphetamine exposure
Source: Retrovirology. 2026 Aug 18;23:14. doi: 10.1186/s12977-026-00684-7 (PMC13757001; doi:10.1186/s12977-026-00684-7)
Supplement: Supplementary file 1 [file 12977_2026_684_MOESM1_ESM.pdf]

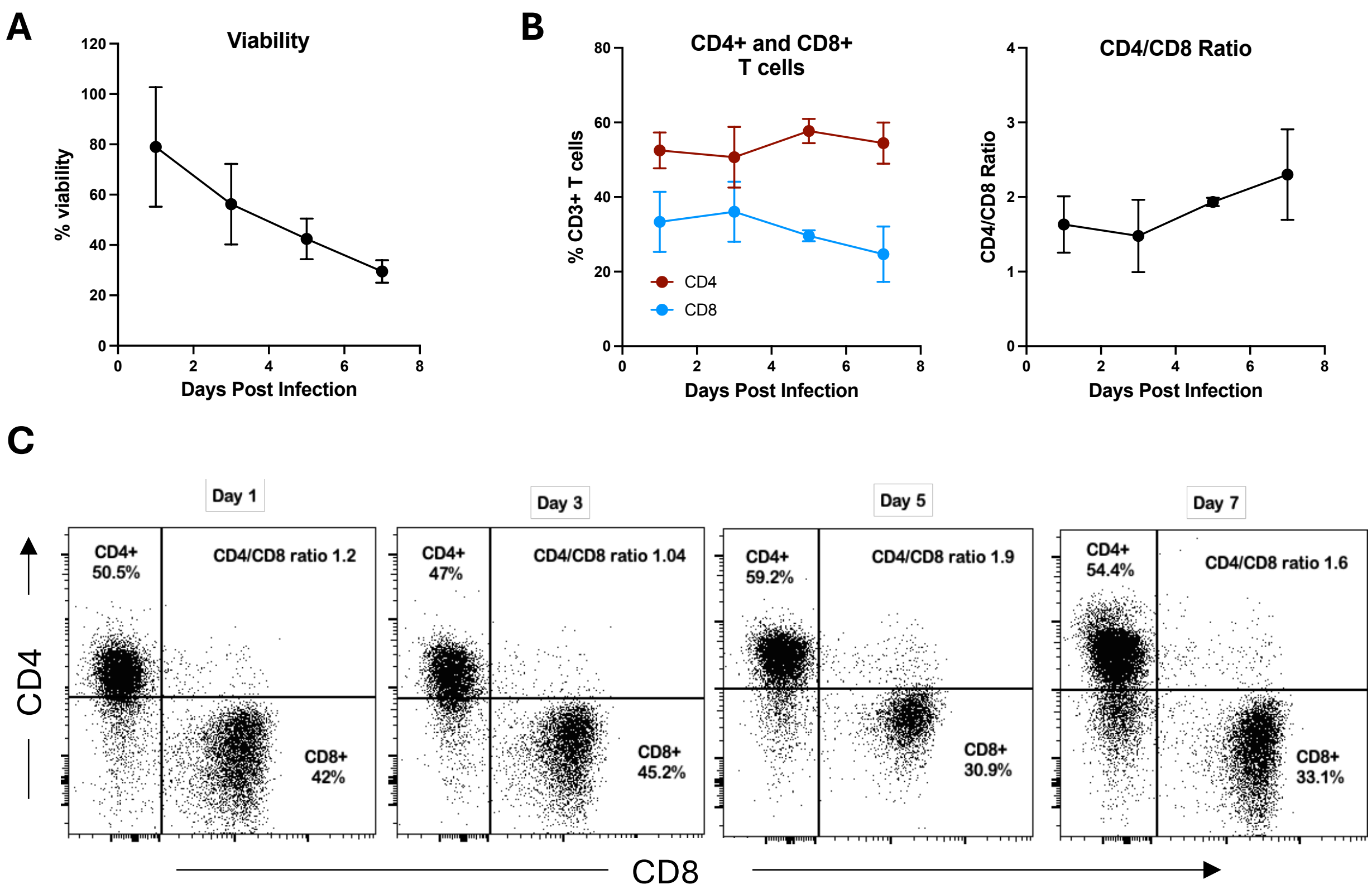

Supplementary Figure 1. Explant mucosal cell viability and T cell dynamics in culture. (A) Viability as measured by trypan blue dye exclusion on isolated mucosal mononuclear cells from cultured colorectal explants. Dots indicate mean of three independent donors with error bars showing standard deviation. **(B)** Percentage of CD4+ and CD8+ T cells out of all CD3+ T cells (left) and CD4/CD8 ratio from these percentages (right) determined by flow cytometry. Dots indicate mean of three independent donors with error bars showing standard deviation. **(C)** Mucosal mononuclear cells from cultured colorectal explants were isolated then stained for flow cytometry with the following antibodies: CD45-PerCP-Cy5.5, CD4-APC, CD8-APC-H7, CD3-Pacific Blue. Percentages were determined using Flow Jo with the following gating: FSC vs SSC → singlets (FSC-A vs FSC-H) → live cells (live/dead stain) → CD45+ cells → CD3+ cells. Dot plots show CD4 vs CD8 percentages of the CD3+ population from one donor.

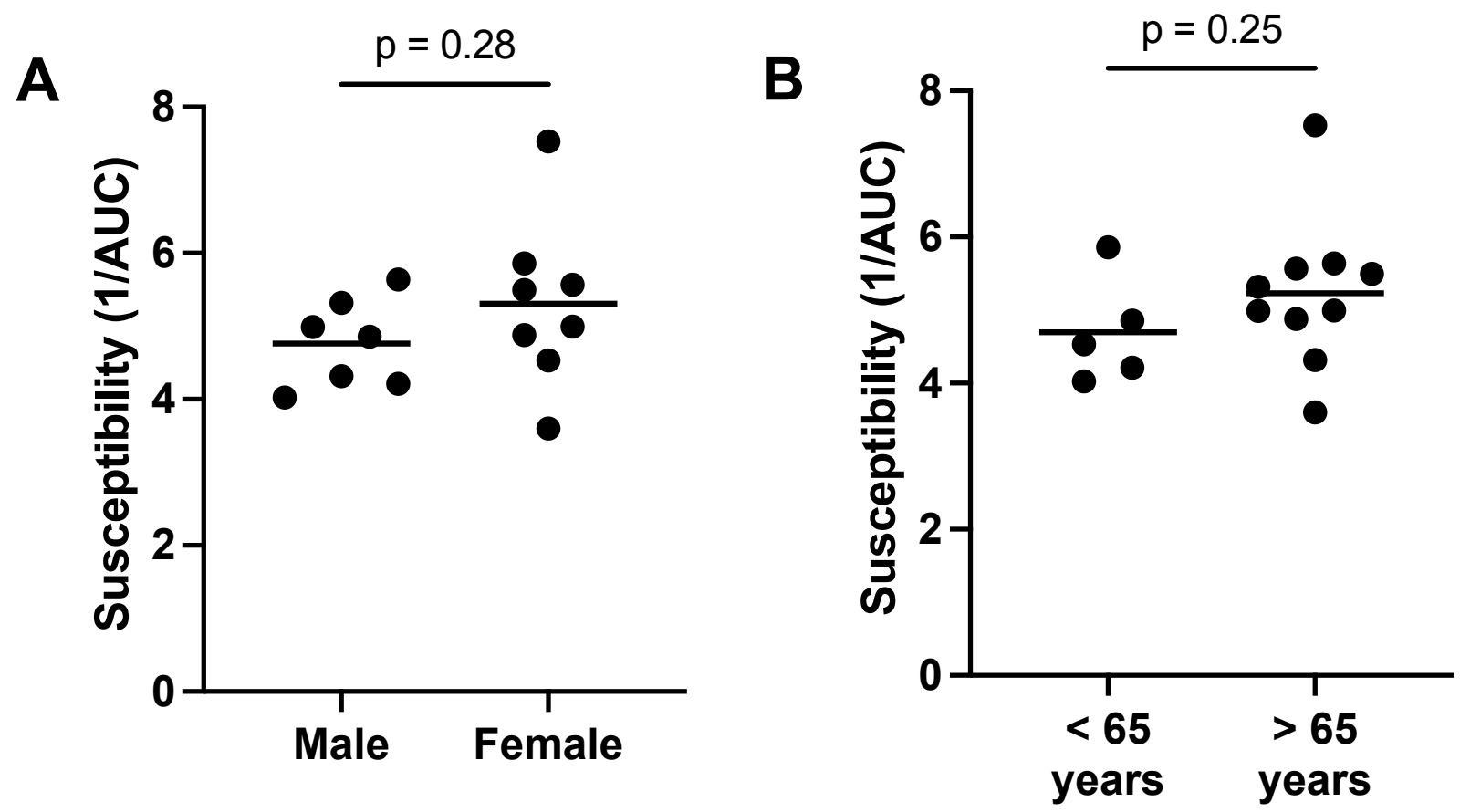

Supplementary Figure 2. Tissue susceptibility comparisons by age and sex. Comparative susceptibility measure using *ex vivo* explant susceptibility assay by **(A)** sex or **(B)** age. Each point represents an individual donor. P-value determined by Mann-Whitney tests.
